# Methods for the isolation and 3D culture of dermal papilla cells from human hair follicles

**DOI:** 10.1111/exd.13368

**Published:** 2017-06-13

**Authors:** Helena Topouzi, Niall J. Logan, Greg Williams, Claire A. Higgins

**Affiliations:** ^1^ Department of Bioengineering Imperial College London London UK; ^2^ Farjo Hair Institute London UK

**Keywords:** dermal papilla, hair follicle, inversion, micro‐dissection, spheroid culture

## Abstract

The dermal papilla is a cluster of mesenchymal cells located at the base of the hair follicle which have a number of important roles in the regulation of hair growth. As a consequence, in vitro models of these cells are widely used to study the molecular mechanisms which underlie hair follicle induction, growth and maintenance. While dermal papilla from rodent hair follicles can be digested prior to cell isolation, the unique extracellular matrix composition found in human dermal papilla renders enzymes such as trypsin and collagenase insufficient for digestion of the dermal papilla into a single cell suspension. As such, to grow human dermal papilla cells in vitro, the papilla has to first be isolated via a micro‐dissection approach from the follicle. In this article we describe the micro‐dissection and culture methods, which we use within our laboratory, for the study of human dermal papilla cells.

## 1 INTRODUCTION

Hair follicle morphogenesis is driven by reciprocal epithelial‐mesenchymal interactions, with the initiating signal for these interactions thought to arise in the mesenchymal dermis.^1^ With parallels to development, the hair follicle mesenchyme in adult skin plays an important role regulating hair cycle transitions, specifically the initiation of the hair growth phase known as anagen, and exit from the resting phase telogen.^2^ In an anagen hair follicle, the mesenchyme is constituted of two components: the dermal papilla, a flame shaped structure at the base of the follicle, and the dermal sheath, which wraps around the outside of the follicle. While there is movement of cells between the dermal papilla and dermal sheath,^3^, ^4^ the dermal papilla is often the more studied component of the mesenchyme. Not only does it have a role in the initiation of anagen,^2^ but also its placement subjacent to the hair matrix during anagen means it can signal to these cells directing their differentiation into the different lineages of the follicle.^5^, ^6^

In androgenetic alopecia, more commonly known as male pattern baldness, the hair follicle goes through a process known as miniaturisation^7^ during which time its hair shaft becomes smaller.^8^ This reduction in the size of the hair shaft is coupled with a reduction in the size of the dermal papilla,^9^ which is relevant as the volume of the dermal papilla in whisker follicles is believed to be proportional to the volume of the hair shaft.^10^ In androgenetic alopecia, follicles can also become “stuck” in telogen and are unable to re‐enter anagen. While it is known that hair follicle stem cells in miniaturising follicles have a reduced ability to convert into stem cell progenitors,^11^ it is not known whether this is due to a lack of signalling from the dermal papilla, or an inability of these cells to respond to signals from the dermal papilla. However, given the importance of the dermal papilla in the initiation and maintenance of anagen, a number of research groups have used in vivo, ex vivo and in vitro models of the dermal papilla to elucidate the key signals within it which control hair growth. This includes transcriptome analysis on dermal papilla, and models assessing the role of the dermal papilla in the regulation of the hair cycle, hair development and specification of hair type.^12^, ^13^, ^14^, ^15^, ^16^

It has been long reported that intact dermal papillae micro‐dissected from species such as the rat and guinea pig are able to induce new hair and follicle growth in recipient epithelium after transplantation.^17^, ^18^ However, it was not until the 1980s that dermal papilla cells were successfully isolated from rat whisker follicles for growth in vitro.^19^ This was followed by a demonstration showing these cells from rat could induce new hair growth in recipient epithelium even after they were grown in culture.^20^, ^21^ Later, a similar method utilising a micro‐dissection approach was used to isolate and culture human dermal papilla cells.^22^ However, while fully intact human papilla can induce new hair growth,^23^ cells cultured from these dermal papillae are notably dissimilar to their rodent counterparts and quickly lose their inductive capacity with culture.^24^ In their natural environment in vivo, the dermal papilla is found at the base of the hair follicle containing cells surrounded by a proteoglycan‐rich extracellular matrix.^25^ As mentioned above, human dermal papilla cells lose their ability to induce new hair growth in culture. We previously demonstrated that growth of human dermal papilla cells in hanging drops can promote formation of dermal spheroids, which are morphologically akin to intact papilla.^26^ This forced aggregation enables a partial restoration of their inductive memory, and human dermal papilla cells grown in 3D spheroids can induce new hair growth.^24^, ^27^

Nowadays, enzymatic digestion followed by fluorescence‐activated cell sorting (FACS) is frequently used for isolation of murine pelage papilla,^28^ which are too small to be micro‐dissected. However, the unique extracellular matrix composition of human dermal papillae^25^, ^29^ renders them indigestible to a single cell suspension with commonly used enzymes such as trypsin and collagenase. Digestion of the human hair follicle with collagenase will digest everything except the dermal papilla, which remains intact, with only a minor disruption to its extracellular matrix.^30^ As such, a variety of micro‐dissection approaches and explant culture methods are still used to isolate dermal papilla from human scalp follicles.^31^, ^32^ In this article, we provide a detailed methodology on the inversion micro‐dissection technique which we use within our laboratory to isolate dermal papilla cells from human scalp follicles in anagen, as well as some tips on how to avoid common pitfalls. In addition to this, we also detail a method for dermal papilla 3D spheroid formation. Previously, low binding plates or plates with synthetic membranes have been used to generate dermal papilla spheroids;^27^, ^33^ however, here we describe a hanging drop methodology which we use within our laboratory.^26^

## 2 TECHNIQUES

### 2.1 Isolation of human dermal papillae

To obtain human tissues, appropriate ethical approvals should be in place. We use skin samples from patients who have given their informed consent using ICREC‐approved consent forms. Tissue is stored under Human Tissue Authority licence 12275 of the Imperial College Human Tissue Bank. Skin biopsies are often full thickness and therefore have an epidermis, dermis and dermal white adipose tissue, with the base of the hair follicles located in the adipose tissue. If the biopsy is from the scalp, then approximately 90% of these follicles will be in anagen.^34^ The dermal papilla can be identified using a stereomicroscope at a high magnification (Leica M80 on a TL 5000 ergo base). As the dermal papilla is engulfed within the hair matrix, we use an inversion technique for isolation. This dermal papilla cell isolation method can be divided into the following three parts, which are also shown in a Video S1.

#### 2.1.1 Part 1: Cleaning and end‐bulb isolation

**Figure 1:**
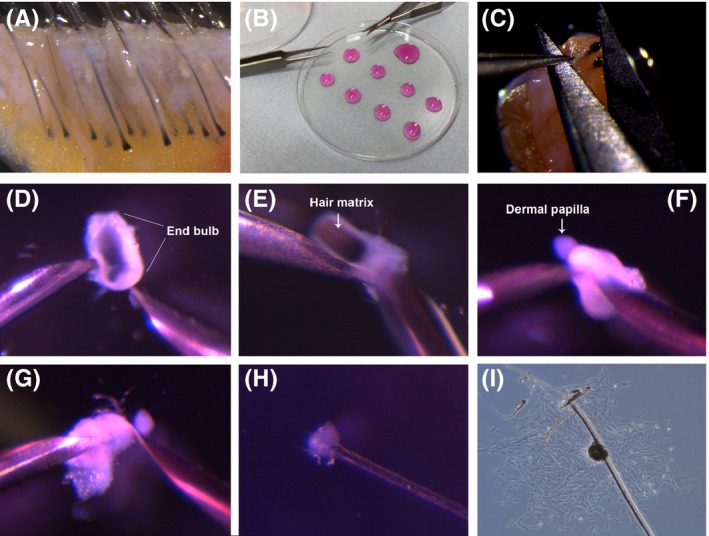
Human dermal papilla micro‐dissection method. (A) Skin biopsy prior to papilla isolation. (B) Petri dish lid with drops of DMEM for dissection. (C) Transection of the hair follicle with scissors. (D) A transected hair follicle end bulb. (E) Inversion of the end bulb. (F) Inverted end bulb and exposed dermal papilla. (G) Transection of the dermal papilla. (H) Dermal papilla adhered to a 35mm dish with a scratch. (I) Growth of cells in an starburst formation around a single dermal papilla.
- While the tissue is washing, prepare plates for use later in the protocol. Using a sterile Pasteur pipette, put eight small drops, and one larger drop of DMEM supplemented with 1% ABAM onto the inverted lid of a petri dish (Figure 1B). Each small drop will be used to hold a single end bulb for inversion in Part 2. Cover these drops by placing the base of the petri dish inside the lid.
- If required, section the tissue into manageable pieces using a sterile scalpel. However, be careful not to cut any hair follicles in half. Using sterile Noyles spring scissors and Dumont forceps, trim away any adherent adipose or connective tissue surrounding the lower section of the follicle to expose the end bulb located at the base of the follicle. The forceps can be used to gently pull up connective tissue and adipose tissue into a peak, away from each follicle, while the scissors can be laid parallel to each follicle to clean efficiently. Tip 1: Cleaning may not always be required if the biopsy contains exposed follicles, as shown in the Video S1.
- Using sterile Dumont straight tip forceps, secure the hair follicle in place so the dermal papilla and hair matrix are visible. Using a pair of scissors, transect the follicle through the matrix just above the papilla to isolate the end bulb (Figure 1C). Carefully transfer the end bulb on the end of the scissors to a clean petri dish containing drops of DMEM/1%ABAM that was prepared in Part 1B (Figure 1B). Drops of DMEM are placed on the lid of the petri dish rather than the base to ensure the optimal angle of inversion, as described in Part 2. After cutting the number of end bulbs required, proceed to the inversion step. Note 1: To establish a culture in a 35‐mm dish, we usually use eight end bulbs distributed in eight drops of DMEM. The larger remaining drop of DMEM is used to collect inverted end bulbs together before isolation of papillae.

- Prior to isolation, autoclave surgical instruments to ensure sterility. Wash the skin biopsy for 10 minutes in a petri dish containing Dulbecco's minimal essential medium (DMEM; Gibco 61965‐026) with 2% antibiotics‐antimycotics (ABAM; Gibco 15240‐062). Following this, transfer the skin biopsy to a new petri dish containing DMEM supplemented with 1% ABAM (Figure 1A).

#### 2.1.2 Part 2: Inversion

- At this point, the end bulbs have a short test tube like shape (a cylinder with a round bottom). The methods below are described for a right‐handed dissector. Left‐handed dissectors may like to use the tools as described or switch hands.
- With a fine needle (27Gx3/4”) held in a 1‐mL syringe in ones left hand, the end bulb should be secured in place by pressing against the cylindrical cut top, on its left side (Figure 1D). Using a second needle (27G×3/4’’) in a 1‐mL syringe in ones right hand, push through the round bottom of the end bulb to invert the structure (Figure 1E) and expose the hair matrix and the dermal papilla residing within (Figure 1F). Tip 2: When inverting the bulb, refrain from holding the needles at a steep angle. Instead, hold the needles as close to the horizontal plane as possible. Tip 3: Make sure to not disrupt or block the opening of the cylinder when the end bulb is being secured in place, as the dermal papilla may not have enough space to invert through the opening.
- At this point, the inverted end bulb should be on the end of the right‐hand needle (Figure 1F). Using the left‐hand needle, pull off the attached matrix. The matrix may have become dislodged during the inversion and if this is the case proceed directly to Part 2H.
- Using the left‐hand needle, gently prize the inverted end bulb off the right‐hand needle by rumpling up the dermal sheath (Figure 1F). This should result in the inverted sheath with the exposed dermal papilla sitting freely in the drop of media. Hold the sheath with the left‐hand needle and gently brush the dermal papilla with the right‐hand needle to remove any remaining matrix cells. Tip 4: Take care when removing the inverted end bulb from the end of the right needle as the exposed dermal papilla can be drawn back within the end bulb if the needle and bulb are attached.
- Transfer the inverted end bulb with the exposed and cleaned dermal papilla to the larger collection drop, which is at the top of the inverted petri dish lid (Figure 1B). Repeat steps E‐I (Part 2) until the required number of inverted end bulbs are contained within the large drop of DMEM. Transection of dermal papillae is performed after collection of all end bulbs, as individual bulbs can be easily lost due to their small size. Tip 5: Ensure that the inverted end bulbs are pushed to the bottom of the drop. If they are floating, they will fall to the edge of the drop and be difficult to locate later on.

#### 2.1.3 Part 3: Dermal papilla transection and adhesion

- Once eight inverted end bulbs are lined up within the larger drop of DMEM/1%ABAM, replace the used needles with new clean needles. Securing the inverted sheath in place with the left‐hand needle, use the sharp edge of the right‐hand needle like a knife and separate the dermal papilla from the connective tissue sheath (Figure 1G). The dermal papilla can then be transferred to a 35‐mm petri dish, containing 2.5 mL of DMEM supplemented with 20% foetal bovine serum (FBS; Gibco 16000044) and 1% ABAM. Tip 6: It should be noted that the dermal papilla can be easily lost during this transfer step. To try and minimise loss, dermal papillae can be transferred in the bevel of a clean needle, taking care that they are not drawn off of the surface of the needle as it is removed from the drop. Extra care is required as the dermal papillae will be moved out of the plane of focus of the microscope. Slowly transfer the dermal papillae between petri dishes, rotate the needle 180° while submerged in the new DMEM/20%FBS/1%ABAM containing 35‐mm dish and gently tap the top of the needle with the other needle to dislodge the dermal papillae. Tip 7: It is best to transfer dermal papillae after all have been transected, as this will reduce mislocation of dermal papilla during this step.
- Transfer approximately eight dermal papillae to each 35‐mm dish and disperse evenly on the bottom of the petri dish. Papilla may be floating on the surface of the medium, so use a low magnification to identify all eight papillae before moving onto the next step. Tip 8: If papillae are floating, use the needle edge to push them to the bottom of the dish. To secure each dermal papilla to the dish, use the rear of the left‐hand needle tip to steady the papilla in place, while simultaneously using a right‐hand needle to scratch through the papilla, adhering it to the base of the plate (Figure 1H). Tip 9: Too much pressure during the scratch will cut the dermal papilla into two, and it will float away. Instead, stub it and then release the pressure and scratch. Note 2: A new set of needles should be used for performing scratches.
- Transfer the 35‐mm plates containing the isolated dermal papillae to an incubator set at standard culture conditions of 37°C/5%CO_2_ in a humidified environment. Tip 10: Care must be taken when transferring plates, as dermal papillae can easily become dislodged. Incubate the plates undisturbed for 10 days, during which time, the papillae collapse and cells grow from each papilla in an explant (Figure 1I). Once the cells are growing well, they can be passaged using standard cell culture techniques subculturing at a 1:2 ratio. Culture medium beyond this point can be changed to DMEM supplemented with 10% FBS and 1% penicillin streptomycin (Gibco 15070063).

### 2.2 Dermal papilla spheroid culture

One limitation with culturing human dermal papilla cells to study hair inductivity is that they lose their inductivity quickly in culture.^24^ However, when dermal papilla cells are grown in 3D dermal spheroids structures, they partially regain their intact transcriptional signature and associated inductivity.^24^ Below, we describe how to prepare 3D spheroids using a hanging drop methodology. For this, we used dermal papilla cells at passage 3‐5, isolated as in the method above. We have divided the procedure into the following two parts:

#### 2.2.1 Part 1: Cell preparation

- Prior to sphere formation, if the dermal papilla cells are too confluent they do not form spheres. Ideally, cells should be harvested for sphere formation once they reach 80% confluence. However, if cells are over confluent, this can be remedied 2 days before spheroid formation. Wash and trypsinise cells as if they are to be regularly passaged. However, instead of passaging at a 50:50 ratio, split the confluent dish at an 80:20 ratio. Keep the dish into which 80% of the cells were seeded for spheroid formation, as described in Part 2C onwards. Tip 1: Passaging cells just 24 hours before spheroid formation can perturb spheroid formation. 48 hours is optimal.
- Dermal papilla cells grown in antibiotics will not form spheres using the hanging drop method. When cells are being passaged as in Part 1A, start using antibiotic‐free medium. If cells are not to be passaged, still change the medium to antibiotic‐free medium at least 48 hours before spheroid culture.

#### 2.2.2 Part 2: Formation of spheroids in hanging drops

**Figure 2:**
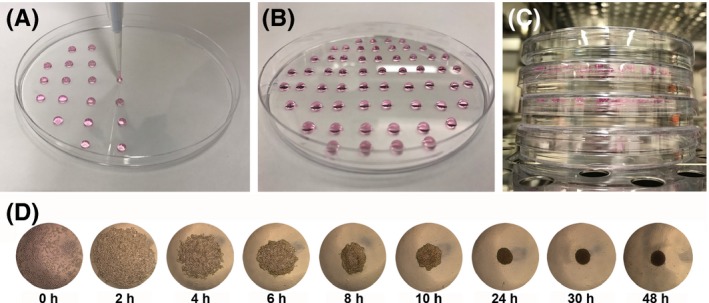
Hanging drop method for spheroid formation. (A) A 20μl pipette placing 10 μl drops, each containing 3000 cells, on the inverted lid of a petri dish. (B) Approximately 60 drops placed on the inverted lid of a 10cm petri dish. (C) Petri dishes containing spheres stacked in an incubator. (D) Bright field images showing sphere formation in a single hanging drop.
- When you have the number of drops needed on the lid (Figure 2B), fill the base of the petri dish with sterile PBS until the bottom is completely covered. Gently turn over the inverted lid containing the cell drops so it is on top of its base. Each drop is now a hanging drop. Note 2: On a 10‐cm petri dish lid, we usually place 60 hanging drops.
- Stack each plate on top of one another. Always have a plate containing PBS with no spheres on the top of the stack (Figure 2C). Note 3: The top petri dish is essential as it maintains a constant temperature in the drops of the top plate. After 24 hours, the cells will have aggregated into a cluster with a spherical structure (Figure 2D).
- The spheroids can be used for experiments between 24 and 48 hours. However, if they are to be kept for longer, the media within the drops will have to be exchanged. At 48 hours, live dead staining can be used to demonstrate spheroid viability. RNA can also be isolated from spheroids for proteomics analysis. Alternatively, they can also be stained as “whole mounts,” or embedded in OCT and sectioned for immunofluorescence analysis.

- When the cells are at approximately 80% confluence, in a Class II laminar flow hood, remove media and wash cells two times with sterile phosphate‐buffered saline (PBS). Remove the PBS and add 0.5% trypsin‐EDTA to cultures before placing cells back in an incubator at 37°C. After approximately 5 minutes, remove the flask from the incubator and using an inverted microscope, check to see whether cells have detached from the flask. Once they have detached, add DMEM/10%FBS to inhibit the trypsin. Transfer the cells in suspension to a 50‐mL Falcon tube and centrifuge for 4 minutes at 200×g to pellet the cells.
- Remove the supernatant and re‐suspend the cell pellet in 10 mL DMEM/10%FBS. Remove 10 μL at this point to count the number of cells on a haemocytometer. Centrifuge the cell suspension again at 200xg for 4 minutes. Note 1: This step ensures that all trypsin is washed off the cells. For normal passaging, cells would only be pelleted and re‐suspended once. However, we find sphere formation is more successful when a second centrifugation step is performed.
- After the second centrifugation, remove all the supernatant. Re‐suspend the pellet in DMEM/10%FBS, at a concentration of 300 cells per microlitre. Tip 2: Use a 1000‐μL pipette to re‐suspend cells, and ensure no bubbles are incorporated into the mix.
- Using a 20‐μL pipette, place 10 μL drops of the 300cell/μL suspension as established in Part 2E, onto the inverted lid of a 10‐cm petri dish (Figure 2A). Each drop will contain 3000 cells, which will aggregate into a dermal papilla spheroid approximately 150 μm in diameter. Tip 3: While pipetting, keep swirling the cells within the falcon tube so they remain in suspension. Tip 4: Hold the pipette perpendicular to the base of the dish for circular drop formation.

## 3 FUTURE PERSPECTIVES

While the skin and hair follicle are perhaps the most accessible tissues in the human body, certainly as compared to other internal tissues and organs, the location of the dermal papilla within the follicle can make isolation by micro‐dissection difficult and time‐consuming. Subsequently, current practice for the isolation of human dermal papilla is commonly reliant upon a skillset in micro‐dissection. New methods of human dermal papilla cell isolation, capable of reducing complexity and time, or increasing cell yield, would be highly advantageous to the skin and hair follicle biology field. The identification of dermal papilla cell‐specific surface markers through the analysis of transcriptome data^16^, ^24^, ^35^ could potentially allow for the use of high‐throughput cell sorting systems, such as FACS or magnetic‐activated cell sorting (MACS). In other reports, dermal fibroblasts from the reticular dermis were specifically sorted using the MACS technique and used for bone regenerative applications.^36^, ^37^ Despite our increased knowledge of papilla‐specific markers, the main limitation with these approaches is that human dermal papilla cannot be digested by commonly used enzymes such as trypsin or collagenase to form viable dermal papilla cells in a single cell suspension. A cocktail of enzymes, capable of digesting the extracellular matrix when the human papilla cells are encapsulated within, while not triggering significant levels of apoptosis could significantly speed up isolation approaches, as waiting for the collapse of the papilla after scratching would no longer be required. If isolation methods were to be improved, larger numbers of intact human dermal papilla would enable a higher number of ex vivo analyses. This is important, as perturbation of cells by culture results in an increase in expression of transcripts associated with cell division, highlighting the unnatural conditions induced by culture. Culture of dermal papilla cells in hanging drops creates a 3D spheroid structure, which more closely resembles the in vivo environment of dermal papilla cells. In terms of cell division, proliferation ceases when papilla cells are grown in spheroids.^26^ We believe this makes spheroid culture a superior model compared to cells in 2D culture, if the questions being asked relate to intact papilla behaviour. This observation, that 3D cell models are superior to 2D cultures, was suggested many years ago in the cancer research field,^38^ and subsequent to this, the use of 3D tumor models has accelerated biomarker and drug discovery.^39^ That being said, the hanging drop method for cell culture can be arduous and time‐consuming. Drug screens require large number of cells to complete, which is not always possible to achieve using primary dermal papilla cells.

To summarise, here we have detailed methods on human dermal papilla isolation, and 3D spheroid culture. The dermal papilla is an indispensable component of the hair follicle that plays a crucial inductive role in hair growth. Isolation and culture of these cells can be arduous and time‐consuming, and dramatic transcriptional shifts occur in culture, decreasing our ability to gain insight to the role of the papilla in hair growth and cycling with in vitro models. Culture of papilla cells in 3D spheroid structures in vitro may provide a useful tool for hair research going forward, and hopefully aid in the discovery of new therapeutic targets for hair growth.

## CONFLICTS OF INTERESTS

The authors have declared no conflicting interests.

## Supporting information


**Video S1** Recorded demonstration of human dermal papilla micro‐dissection.
